# The combination of *Radix Astragali* and *Radix Angelicae Sinensis* attenuates the IFN-γ-induced immune destruction of hematopoiesis in bone marrow cells

**DOI:** 10.1186/s12906-019-2781-4

**Published:** 2019-12-09

**Authors:** Juan Liu, Jie Wei, Changzhi Wang, Xiaoying Meng, Hening Chen, Peiying Deng, Meiyier Huandike, Huijie Zhang, Xue Li, Limin Chai

**Affiliations:** 1 Key Laboratory of Chinese Internal Medicine of Ministry of Education and Beijing, Dongzhimen Hospital, Beijing University of Chinese Medicine, Haiyuncang Hutong No.5, Dongcheng District, Beijing, China; 2 Department of Rheumatology, The Second Affiliated Hospital of GuangZhou University of Chinese Medicine, GuangZhou, China

**Keywords:** *Radix Astragali*, *Radix Angelicae Sinensis*, Immune destruction, Apoptosis, Bone marrow cell

## Abstract

**Background:**

*Radix Astragali* and *Radix Angelicae Sinensis* are two herbs that compose Danggui Buxue Tang (an herbal formula for treatment of anemia diseases). In this study, we explored the molecular mechanism and effective targets to immune destruction of bone marrow (BM) cells treated with *Radix Astragali*, *Radix Angelicae Sinensis* or a combination of two agents. The potential synergic advantages of two herbs should also be explored.

**Methods:**

The constituents of *Radix Astragali* and *Radix Angelicae Sinensis* were analyzed by high performance liquid chromatography-electrospray ionization/mass spectrometer system BM cells were separated from limbs of BALB/c mice, and immune destruction was induced with IFN-γ. The percentages of hematopoietic stem cells (HSCs) and CD3^+^ T cells were detected by flow cytometry. The distribution of T-bet and changes in the combination of SAP and SLAM in BM cells were observed by immunofluorescence. Western blotting was used to assay the expression of key molecules of the eIF2 signaling pathway in BM cells.

**Results:**

Seven constituents of *Radix Astragali* and six constituents of *Radix Angelicae Sinensis* were identified. The percentages of HSCs increased significantly after treatment with *Radix Angelicae Sinensis*, especially at high concentrations. The percentages of CD3^+^ T cells were significantly decreased after *Radix Astragali* and *Radix Angelicae Sinensis* treatment. However, the synergistic function of two-herb combinations was superior to that of the individual herbs alone. The distribution of T-bet in BM cells was decreased significantly after *Radix Angelicae Sinensis* treatment. The number of SLAM/SAP double-stained cells was increased significantly after *Radix Astragali* treatment at low concentrations. The phosphorylation levels of eIF2α were also reduced after *Radix Astragali* and *Radix Angelicae Sinensis* treatment.

**Conclusions:**

*Radix Astragali* and *Radix Angelicae Sinensis* could intervene in the immunologic balance of T lymphocytes, inhibit the apoptosis of BM cells induced by immune attack, restore the balance of the T cell immune response network and recover the hematopoietic function of HSCs. The synergistic effects of *Radix Astragali* and *Radix Angelicae Sinensis* were superior to those of each herb alone.

## Background

The pathophysiology of unusual hematologic diseases is immune mediated with activated type I cytotoxic T cells; that express T cell-specific cytokines, especially γ-interferon (IFN-γ). Immune attack leading to bone marrow failure by T cells can be imitated in vitro. IFN-γ is an important cytokine in orchestrating a vast array of immunologic responses and is also known as a suppressor of hematopoiesis [1]. Several studies have confirmed that IFN-γ can induce apoptosis of hematopoietic stem cells (HSCs), partially through the Fas-dependent apoptotic pathway. Bone marrow (BM) failure in multiple chronic inflammatory diseases is associated with increased IFN-γ levels in the bone microenvironment [2, 3]. IFN-γ can also regulate the physiological function of interferon regulatory factor 1 (IRF-1), inhibit the transcription of cellular genes, and contribute to attenuating the cell cycle of BM cells [4].

Herbal partners through clinical application are based on an affirmance theory foundation and compounding prescript of Chinese traditional medicine. *Radix Astragali* and *Radix Angelicae Sinensis* are a classic pair of available herbs in Chinese medicine for clinical anemia treatment [5–8]. Pharmacological studies indicated that *Radix Angelicae Sinensis* could be used to invigorate the blood circulation, and modulate the balance of the immune system in menstrual disorders, [9]. Several studies indicated that *Radix Astragali* has therapeutic functions including immunostimulation [10], hepatoprotection [11], anti-diabetic effects [12], analgesia [13] and sedation [14]. In addition, Danggui Buxue Tang (DBT, a classic Chinese herbal formula consisting of *Radix Astragali* and *Radix Angelicae Sinensis*) could promote hematopoietic function, stimulate cardiovascular circulation, prevent osteoporosis and has antioxidative functions [15–17]. Our previous studies have verified the hematopoietic function of modified DBT in anaplastic anemia mouse model by bone marrow suppression. The results indicated that modified DBT could decrease the proliferation and differentiation of effector T cells, impair Treg-mediated immunosuppressive functions, attenuate immune-mediated destruction of HSCs, repair hematopoietic failure and recover the hematopoietic function of HSCs in vivo [18, 19].

Based on the in vivo results, we wanted to investigate the functional mechanism of *Radix Astragali*, *Radix Angelicae Sinensis* or the two drugs together on immunosuppressive effects. BM cells induced by increasing doses of IFN-γ were used as a cell model of immune destruction [20]. *Radix Astragali*, *Radix Angelicae Sinensis* or the combination of the two herbs was used to intervene in IFN-γ-induced immune destruction of hematopoiesis of BM cells. We wanted to study the specific cellular and protein targets of the immunosuppressive and hematopoietic functions of *Radix Astragali* and *Radix Angelicae Sinensis* on immune- attacked BM cells, and then probe the potential synergic mechanism of the combination of the two herbs. In this study, we used innovative in vitro experiments to verify the synergistic effect of this Chinese herbal formula.

## Methods

### Preparation of freeze-dried *Radix Astragali* and *Radix Angelicae Sinensis* water extract

*Radix Astragali* (Root pieces, Lot No. 19042102, origin: Inner Mongolia, China) and *Radix Angelicae Sinensis* (Root pieces, Lot No. 19050802, origin: Gansu, China), were purchased from Beijing Xidan Pharmaceutical Co., Ltd., China. Dr. Jie Wei, a senior Chinese medicine appraiser, undertook the formal identification of the herbs. The herbal inspection reports are shown in Additional file 1. We prepared aqueous extracts of the two herbs separately. A total of 200 g of raw herbal pieces was boiled in a 6× volume of water for 30 min. The aqueous extract solution was concentrated to a volume of 100 mL. Finally, the extract solution was filtered using a standard test sieve of 150 μm, freeze-dried and maintained in desiccators at 4 °C until use [21, 22].

### High-performance liquid chromatography-electrospray ionization/ mass spectrometer (HPLC-ESI/MS^n^) analysis

The freeze-dried powders of *Radix Astragali* and *Radix Angelicae Sinensis* water extracts were used for component analysis. The constituents of these two herbs were measured by HPLC-ESI/MS^n^. The specific measurement procedures were previously described [23].

### Obtaining mouse BM cells

Female BALB/c mice (Protocol No. SCKK (Jing) 2016–006) were purchased from HFK Bioscience Co. Ltd. (Beijing, China). All animals were kept under standard lighting conditions (12 h alternating day and night cycles) and given free access to food and water. Animal experiments were performed according to protocols complied with ethical regulations and approved by the National Institute of State Scientific and Technological Commission.

Single-cell suspensions of bone marrow were isolated and cultured. Briefly, eight-week-old female mice were sacrificed by pentobarbital anesthesia (1%, 45 mg/kg). The tibias and femurs were collected in a sterile environment, and the ends of the long bones were trimmed to expose the interior marrow shaft. Bone marrow cells were rinsed with RPMI-1640 (Thermo Fisher Scientific Inc., Waltham, MA, USA) medium supplemented with 10% FBS (Gibco, Grand Island, NY). To make a single-cell suspension, the were gently drawn up and down with a 3-cc syringe with a 21-g needle, filtered through a 70-mm filter mesh, washed and resuspended. Cells were incubated at 37 °C in a 5% CO_2_ incubator [24].

### Cell viability assay

BM cells taken from mice were plated and cultured in a 96-well plate (1 × 10^5^ cells/well) in RPMI-1640 supplemented with 10% FBS, Various concentrations of the water extract freeze-dried powders of *Radix Astragali* or *Radix Angelicae Sinensis* (0, 10, 50, 100, 250, 500, 750 and 1000 μg/mL) were added to the medium, and incubated at 37 °C in a humidified 5% CO_2_ incubator. After 24 h of incubation, cell viability was determined by a Cell Counting Kit-8 (CCK-8) assay according to the manufacturer’s instructions. BM cells (1 × 10^4^/well) were seeded into a 96-well plate and incubated overnight in the previously described conditions. Then, the medium was removed, and the cells were washed twice with PBS. Medium (90 μL) and CCK-8 (10 μL) were subsequently added to each well and incubated for 2 h at 37 °C. The optical density (OD value) of the cells was measured by a microplate reader (Model 3550 Microplate Reader, Bio-Rad Laboratories, Inc., Hercules, CA, USA) at a wavelength of 570 nm [25]. All experiments were performed in triplicate. The results of the CCK-8 assay indicated that 100 μg/mL and 250 μg/mL were suitable concentrations for drug stimulation (Additional file 2: Fig. S1).

### Cell treatments

BM cells were seeded in 6-well plates (1 × 10^6^ cells/well) or 24-well plates (1 × 10^5^ cells/well), and divided into following groups: Normal group (N group), incubated in RPMI-1640 containing 10% FBS; Model group (M group), incubated in RPMI-1640 containing 10% FBS with IFN-γ (10 ng/mL) (R&D systems, USA); *Radix Astragali* group (R group), incubated in RPMI-1640 containing 10% FBS with IFN-γ (10 ng/mL) and freeze-dried powder (*Radix Astragali*) (100 μg/mL or 250 μg/mL); *Radix Angelicae Sinensis* group (A group), incubated in RPMI-1640 containing 10% FBS with IFN-γ (10 ng/mL) and freeze-dried powder (*Radix Angelicae Sinensis*) (100 μg/mL or 250 μg/mL); and the two-herb combination group (R + A group), incubated in RPMI-1640 containing 10% FBS with IFN-γ (10 ng/mL) and freeze-dried powders (*Radix Astragali* and *Radix Angelicae Sinensis*) (100 μg/mL or 250 μg/mL each). After 12 h or 24 h of incubation, the cells were harvested.

### Flow cytometry analysis

To quantify the percentage of hematopoietic stem cells (HSCs), BM cells (1 × 10^5^ cells) were stained with anti-mouse CD117 (c-Kit) FITC and anti-mouse Ly-6A/E (Sca-1) PE antibodies (eBioscience, San Diego, CA, USA). The percentages of CD3^+^CD4^+^ (helper T cell, Th) and CD3^+^CD8^+^ (cytotoxic T cell, CTL) lymphocytes were also measured. After 12 h or 24 h of treatment, BM cells were harvested and stained with anti-mouse CD3 PE-Cyanine7, CD4 PE-Cyanine5 and CD8a PE antibodies (eBioscience, San Diego, CA, USA). The percentages of staining cells were counted and analyzed by FACS Calibur cytometer and CellQuest software (Beckman Coulter, Brea, CA, USA). Each experiment was performed in triplicate with three replicates each.

### Immunofluorescence staining

After 12 h or 24 h of treatment, BM cells were collected. Cells (0.5 × 10^4^ cells/well) were planted in special petri dish for laser confocal microscope, and then incubated with anti- mouse T-bet PE (1:100) (eBioscience). In addition, the other portion of the BM cells was stained with anti-mouse CD150/SLAM PE antibody (1:50) (eBioscience). Cells were stained with SH2D1A/SAP antibody (FITC) (1:100) (EterLife, Birmingham, UK) after permeabilization. A Leitz/Leica TCSSP2 microscope (Leica Lasertechnik GmbH, Heidelberg, Germany) wasused to capture the fluorescence microscopy images. The fluorescence intensity was analyzed by ImageJ software. Each experiment was performed in triplicate with three replicates each.

### Western blotting analysis

After 12 h or 24 h of treatment, BM cells (1 × 10^6^ cells) were washed three times with PBS and collected in RIPA lysis buffer. Total proteins were separated by 10% sodium dodecyl sulfate (SDS)-polyacrylamide gel electrophoresis (PAGE) and transferred onto nitrocellulose membranes (Amersham Pharmacia Biotech, Uppsala, Sweden). Specific antibodies were used to measure the levels of proteins, including anti-mouse eIF2α and phospho-eIF2α rabbit monoclonal antibodies (1:1000) (CST, Boston, MA, USA). The nitrocellulose membranes were incubated with specific antibodies at 4 °C overnight. Next, the membranes were incubated with a horseradish peroxidase-conjugated secondary antibody (CST). Immunoreactive proteins were visualized by the enhanced ECL method (Amersham Biosciences, Marlborough, MA), and β-actin was used as an internal control. Each experiment was performed in triplicate with three replicates each. Densitometry values were quantified for each band using Image-Pro Plus version 4.0 (Media Cybernetics, Rockville, MD, USA).

### Statistical analysis

All data are presented as the mean ± standard deviation (SD) of the mean from at least three independent experiments. GraphPad Prism 5.0 and SPSS version 13.0 were used for data processing and statistical analysis. A *P* < 0.05 was considered statistically significant.

## Results

### Characteristics of compounds in freeze-dried water extract by HPLC-ESI/MS^n^

**Fig. 1:**
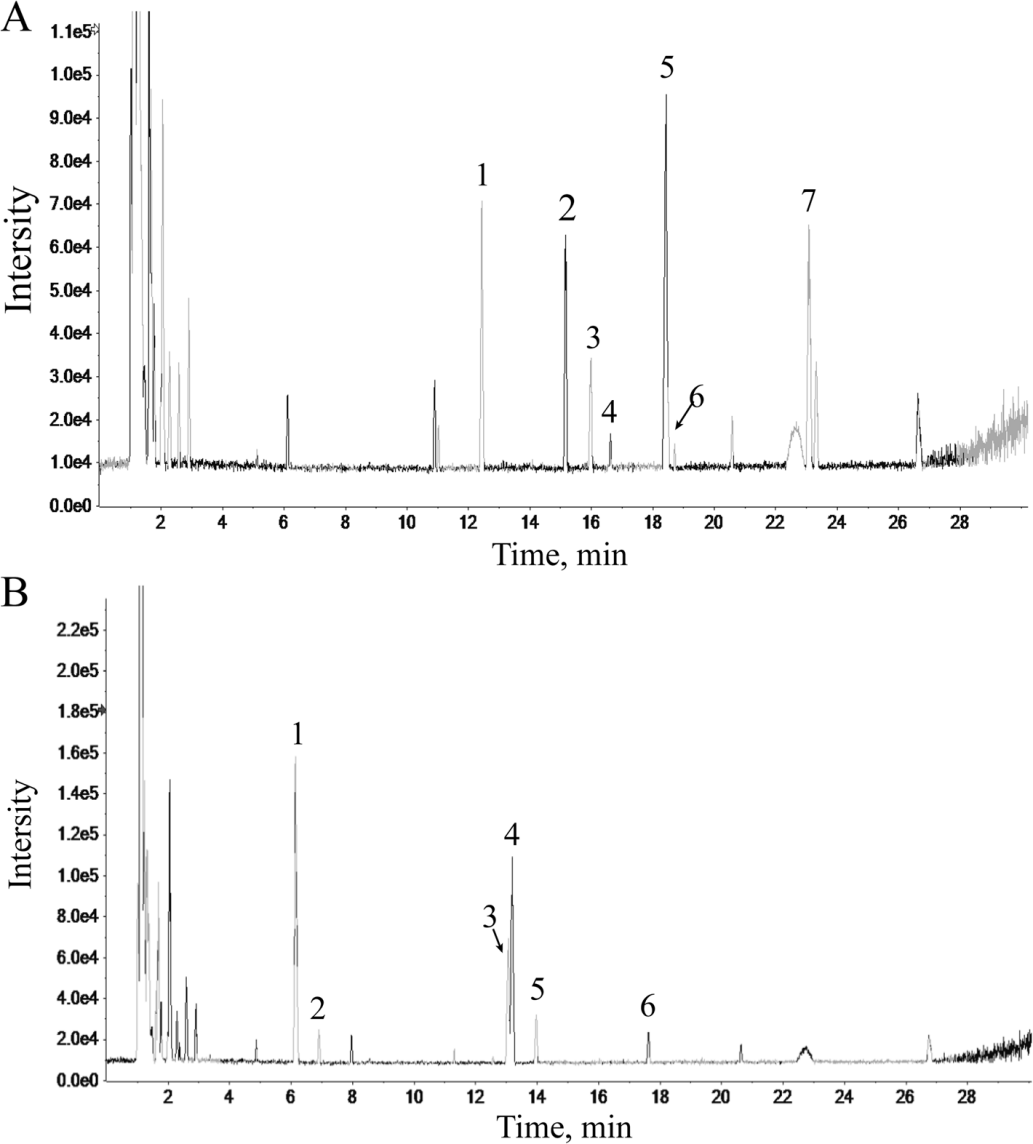
HPLC-ESI/MS^n^ base peak chromatogram of the total ion chromatogram for the lyophilized powder of *Radix Astragali* and *Radix Angelicae Sinensis* aqueous extract. The abscissa represents the retention time, and the ordinate represents the chromatographic peak intensity. (**a**) HPLC-ESI/MS^n^ analysis of *Radix Astragali*. (**b**) HPLC-ESI/MS^n^ analysis of *Radix Angelicae Sinensis*

Seven constituents of *Radix Astragali* (Fig. 1a) and six constituents of *Radix Angelicae Sinensis* (Fig. 1b) were identified based on the target peaks. The identified compounds in the freeze-dried powders of aqueous extracts from these to herbs are shown in Tables 1 and 2.

**Table 1:** Chemical components identified from Radix Astragali by HPLC-ESI/MS^n^

Peak	t_R_ (min)	Identification
1	12.437	Calycosin-7-*O*-β-D-glucoside
2	15.161	Calycosin-7-*O*-β-D-glucoside-6*″*-*O*-malonate
3	15.979	Ononin
4	16.624	(6a*R*,-11a*R*)-3-Hydroxy-9,10-dimethoxypterocarpan-3-*O*-β-D-glucoside
5	18.416	Calycosin
6	18.498	Formononetin-7-*O*-β-D-glucoside-6″-*O*-malonate
7	23.087	Formononetin

**Table 2:** Chemical components identified from Radix Angelicae Sinensis by HPLC-ESI/MS^n^

Peak	t_R_ (min)	Identification
1	6.14	L-tryptophan
2	6.909	Vanillic acid
3	13.06	Ferulic acid
4	13.2	Senkyunolide I
5	13.973	Senkyunolide H
6	17.623	Z (or E)-Butylidenephthalide

### Effects of *Radix Astragali*, *Radix Angelicae Sinensis* and the combination of both on the proliferation of HSCs in IFN-γ-induced BM cells

**Fig. 2:**
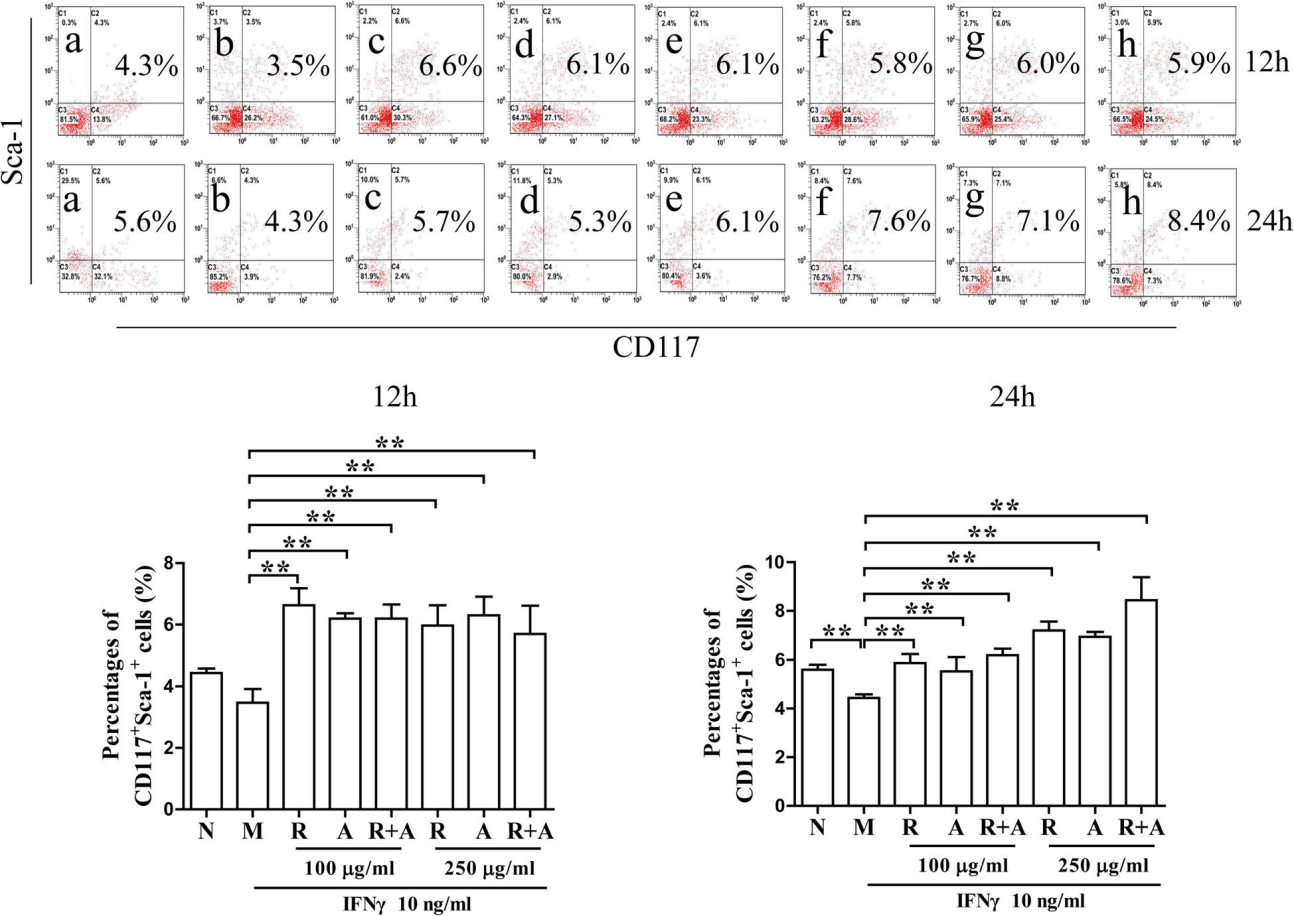
Effects on the proliferation of CD117^+^Sca-1^+^ HSCs in IFN-γ -induced BM cells after 12 and 24 h of treatment. The results are presented in bar charts. a, Normal (N) group; b, Model (M) group; c and f, *Radix Astragali* (R) group; d and g, *Radix Angelicae Sinensis* (A) group; e and h, *Radix Astragali* + *Radix Angelicae Sinensis* (R + A) group; c, d and e were treated with 100 μg/mL of freeze-dried powder and f, g and h were treated with 250 μg/mL of freeze-dried powders. The results are presented in a bar chart. Data are the presented as mean ± SD, *n* = 3. *P < 0.05 and **P < 0.01

CD117 (c-Kit) and Ly-6A/E (Sca-1) are the cell membrane markers for HSCs [26]. Cells double-stained by CD117 and Sca-1 were assessed through flow cytometry. As shown in Fig. 2, the percentages of HSCs in the model group after 12 h or 24 h of induction by IFN-γ decreased significantly compared with those in the normal group (*P* < 0.01). The percentages of HSCs in the treatment groups were increased significantly compared with those in the model group after 12 h or 24 h of treatment (*P* < 0.01). There was no significant difference between the two concentrations (100 μg/mL and 250 μg/mL) of herbs after 12 h of treatment. Interestingly, the percentage of HSCs in the 250 μg/mL concentration group after 24 h of treatment increased significantly more than that of the other groups. These results indicate that the synergistic effect of the combination of the two herbs on hematopoiesis was superior to that of a single herb alone. This effect showed significant time and dose dependence.

### The proliferation and differentiation of CD3^+^ T cells in BM cells after treatment

**Fig. 3:**
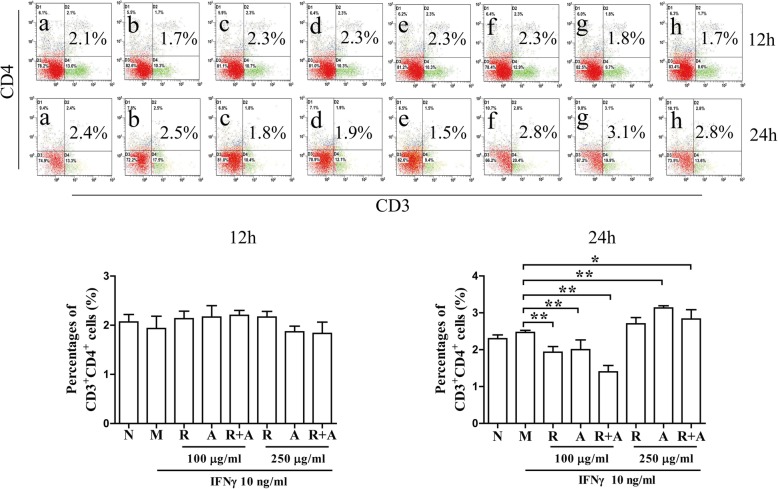
Effects on the proliferation of CD3^+^CD4^+^ T cells in BM cells after 12 and 24 h of treatment. The results are presented in bar charts. a, Normal (N) group; b, Model (M) group; c and f, *Radix Astragali* (R) group; d and g, *Radix Angelicae Sinensis* (A) group; e and h, *Radix Astragali* + *Radix Angelicae Sinensis* (R + A) group; c, d and e were treated with 100 μg/mL of freeze-dried powders, and f, g and h were treated with 250 μg/mL of freeze-dried powders. The results are presented in a bar chart. Data are presented as the mean ± SD, *n* = 3. *P < 0.05 and **P < 0.01

**Fig. 4:**
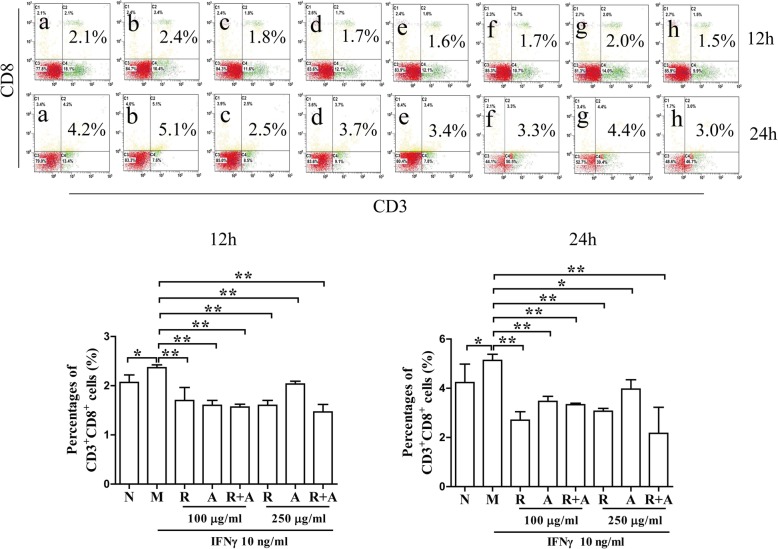
Effects on the proliferation of CD3^+^CD8^+^ T cells in BM cells after 12 and 24 h of treatment. The results are presented in bar charts. a, Normal (N) group; b, Model (M) group; c and f, *Radix Astragali* (R) group; d and g, *Radix Angelicae Sinensis* (A) group; e and h, *Radix Astragali* + *Radix Angelicae Sinensis* (R + A) group; c, d and e were treated with 100 μg/mL of freeze-dried powders, and f, g and h were treated with 250 μg/mL of freeze-dried powders. The results are presented in a bar chart. Data are presented as the mean ± SD, *n* = 3. *P < 0.05 and **P < 0.01

As shown in Fig. 3, the percentages of CD3^+^CD4^+^ T cells were not significantly different among the groups after 12 h of treatment. The levels of cellular proliferation in the treatment groups after 24 h of treatment were significantly higher than those in the other groups (*P* < 0.05 or *P* < 0.01). However, treatment with RA or RAS decreased the IFN-γ-induced abnormal proliferation of CD3^+^CD8^+^ cytotoxic T cells in BM cells. The inhibitory effect of *Radix Astragali* was better than that of *Radix Angelicae Sinensis*, and the synergistic effect of the combination of the two herbs was superior to that of a single herb alone (Fig. 4).

### Regulation of the SLAM/SAP signaling pathway activation by herbal treatment

**Fig. 5:**
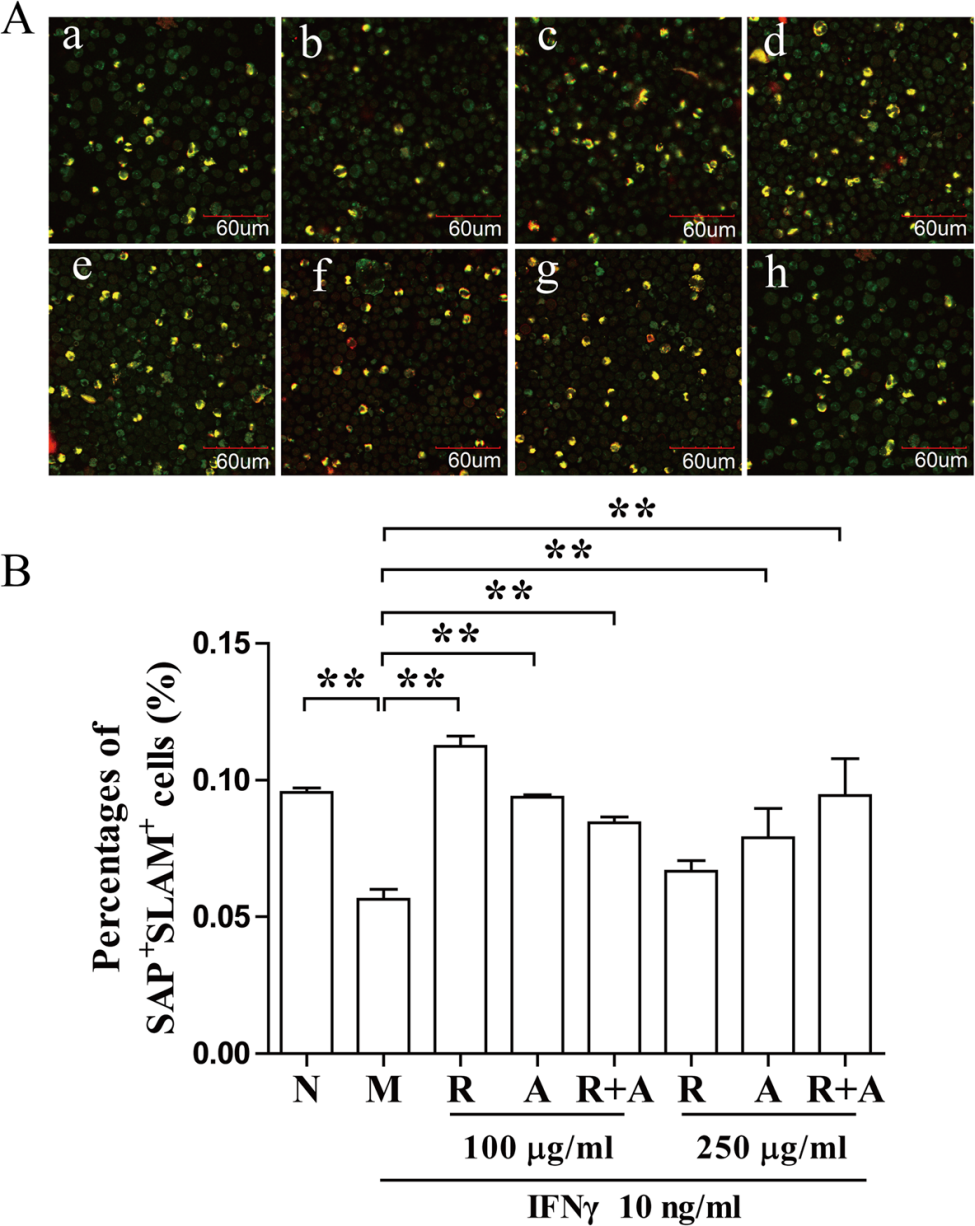
Regulation of activation of the SLAM/SAP signaling pathway after 12 h of treatment. (**a**) BM cells were stained with CD150/SLAM and SH2D1A/SAP antibodies and observed by confocal immunofluorescence microscopy. The double-stained cells (yellow) were quantified with ImageJ. The scale bar corresponds to 60 μm throughout. (**b**) The quantified results are presented in a bar chart. a, Normal (N) group; b, Model (M) group; c and f, *Radix Astragali* (R) group; d and g, *Radix Angelicae Sinensis* (A) group; e and h, *Radix Astragali* + *Radix Angelicae Sinensis* (R + A) group; c, d and e were treated with 100 μg/mL of freeze-dried powders, and f, g and h were treated with 250 μg/mL of freeze-dried powders. Data are presented as the mean ± SD, *n* = 3. *P < 0.05 and **P < 0.01

**Fig. 6:**
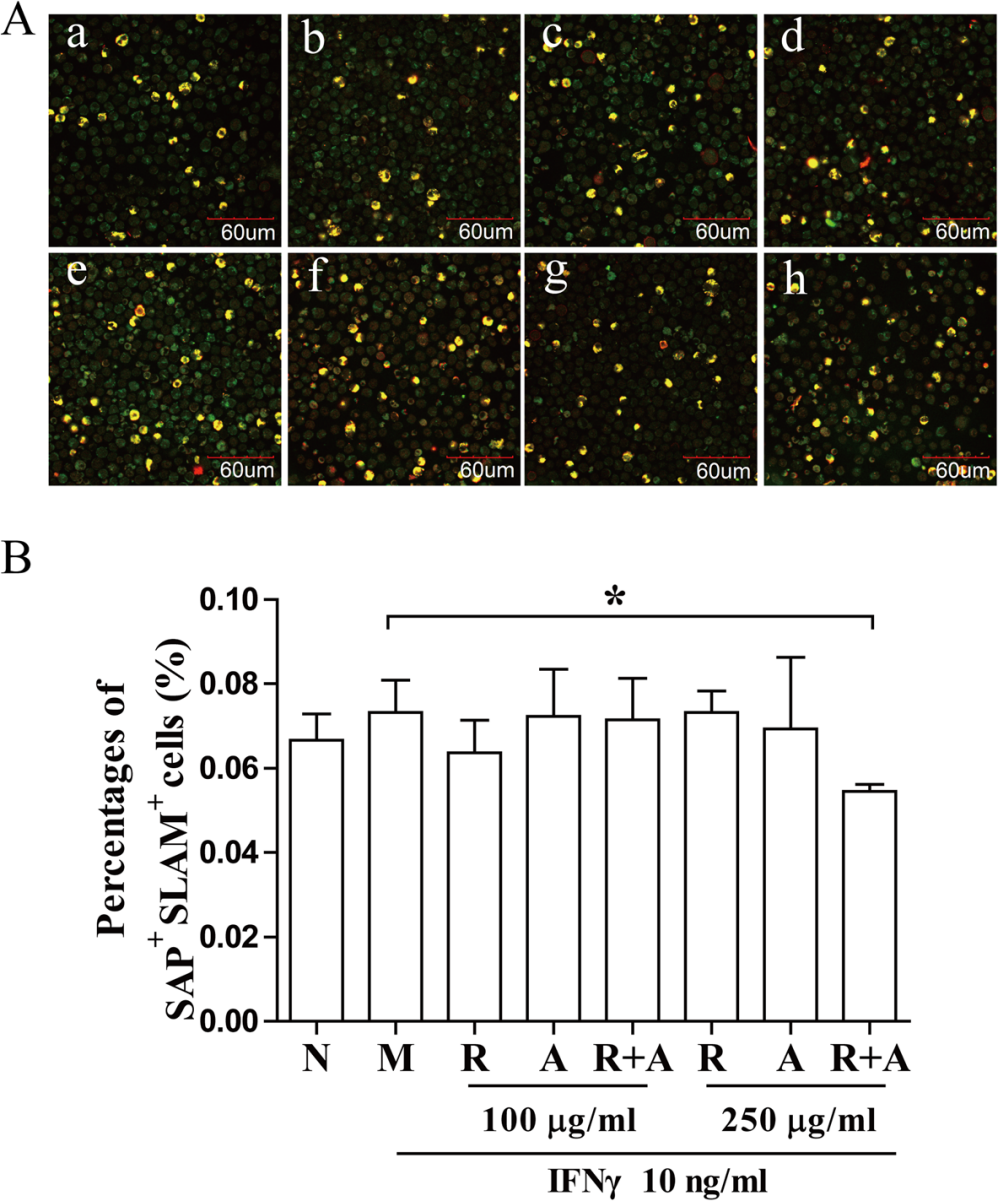
Regulation of activation of the SLAM/SAP signaling pathway after 24 h of treatment. (**a**) BM cells were stained with CD150/SLAM and SH2D1A/SAP antibodies and observed by confocal immunofluorescence microscopy. The double-stained cells (yellow) were quantified with ImageJ. The scale bar corresponds to 60 μm throughout. (**b**) The quantified results are presented in a bar chart. a, Normal (N) group; b, Model (M) group; c and f, *Radix Astragali* (R) group; d and g, *Radix Angelicae Sinensis* (A) group; e and h, *Radix Astragali* + *Radix Angelicae Sinensis* (R + A) group; c, d and e were treated with 100 μg/mL of freeze-dried powders, and f, g and h were treated with 250 μg/mL of freeze-dried powders. Data are presented as the mean ± SD, *n* = 3. *P < 0.05 and **P < 0.01

The activation of SLAM signaling pathway contribute to the proliferation and differentiation of T cells. The binding of SLAM and SAP transmits signaling to Fyn, inhibits the production of IFN-γ in BM cells [27]. After 12 h of treatment, SAP/SLAM double-stained cells were significantly increased in the treatment groups compared with those in the model group (*P* < 0.01) (Fig. 5). The regulatory effect of *Radix Astragali* in the 100 μg/mL concentration group after 12 h of treatment increased significantly more than the in other groups. However, there was no significant difference between the groups after 24 h of treatment (Fig. 6).

### Interference in the expression and distribution of T-bet in BM cells by herbal treatment

T-bet is the Th1-specific transcription factors. It binds to the gene promoter of IFN-γ, contributes to the IFN-γ gene expression [28]. As shown in . 7, T-bet-stained cells were significantly increased in BM cells of the model group compared with that in the normal group after 12 h of treatment (*P* < 0.05). Both *Radix Astragali* and *Radix Angelicae Sinensis* significantly inhibited the IFN-γ-induced expression and distribution of T-bet in BM cells. Interestingly, the distribution of T-bet in the combination groups after 24 h of treatment decreased significantly more than in the other groups (*P* < 0.01) (Fig. 8). These results indicate that the synergistic effect of the two-herb combination was superior to that of herb alone, and showed significant time and dose dependence.

**Fig. 7:**
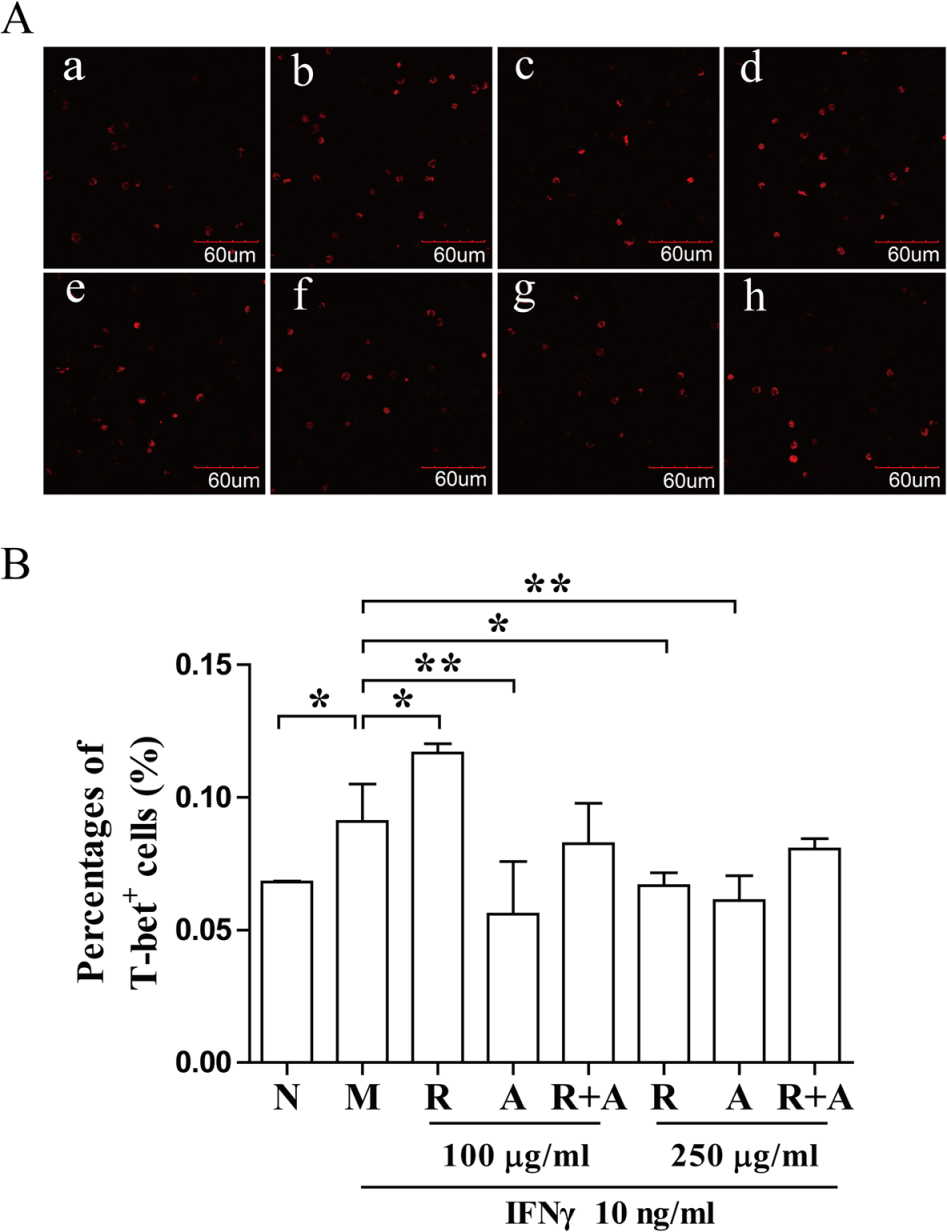
Interference in the expression and distribution of T-bet in BM cells after 12 h of treatment. (**a**) Staining for T-bet (red) in BM cells was observed by confocal immunofluorescence microscopy. The stained cells (red) were quantitated with ImageJ. The scale bar corresponds to 60 μm throughout. (**b**) The quantified results are presented in a bar chart. a, Normal (N) group; b, Model (M) group; c and f, *Radix Astragali* (R) group; d and g, *Radix Angelicae Sinensis* (A) group; e and h, *Radix Astragali* + *Radix Angelicae Sinensis* (R + A) group; c, d and e were treated with 100 μg/mL of freeze-dried powders and f, g and h were treated with 250 μg/mL of freeze-dried powders. Data are presented as the mean ± SD, *n* = 3. *P < 0.05 and **P < 0.01

**Fig. 8:**
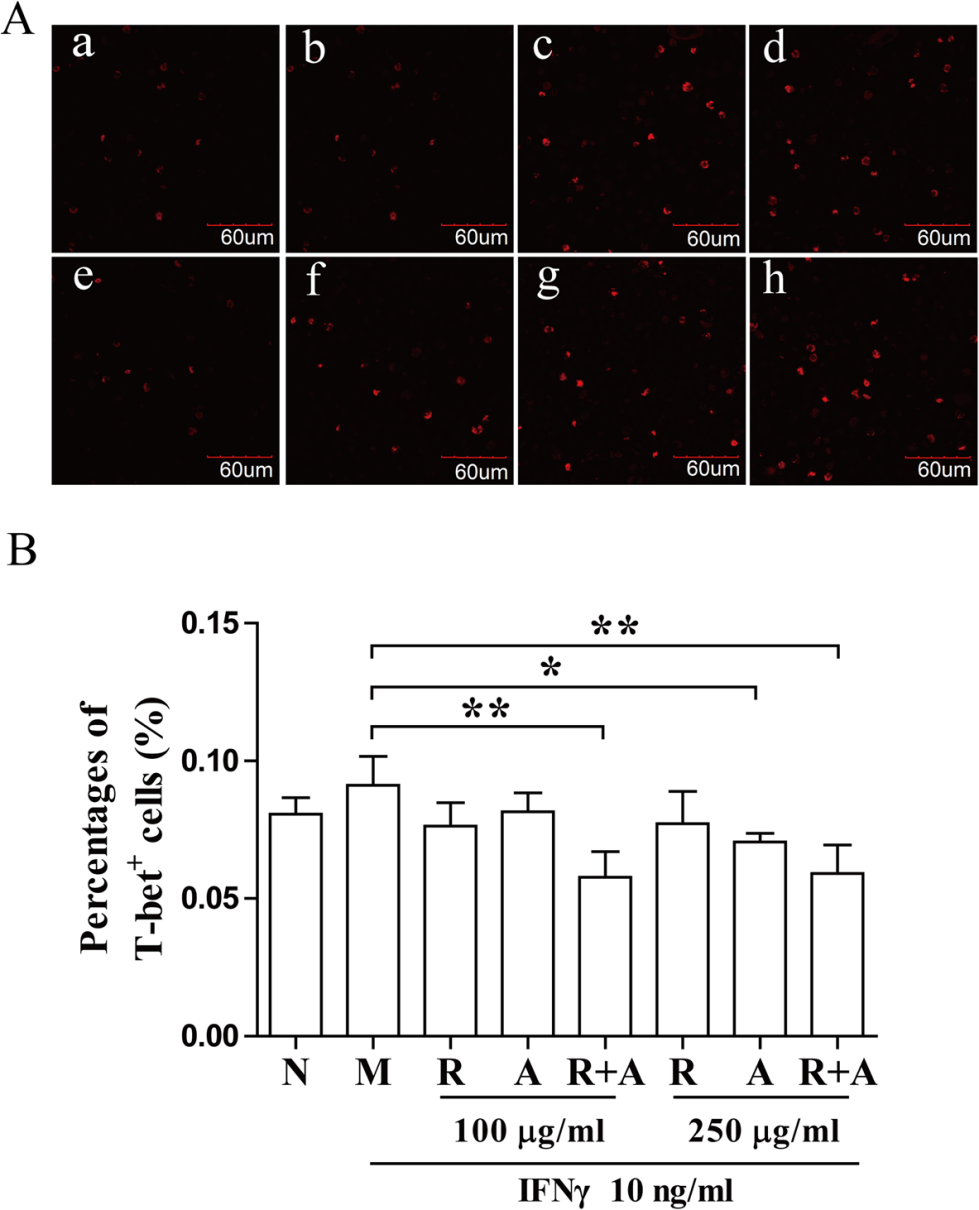
Interference in the expression and distribution of T-bet in BM cells after 24 h of treatment. Staining for T-bet (red) in BM cells was observed by confocal immunofluorescence microscopy. The stained cells (red) were quantitated with Image J. The scale bar corresponds to 60 μm throughout. (**b**) The quantified results are presented in a bar chart. a, Normal (N) group; b, Model (M) group; c and f, *Radix Astragali* (R) group; d and g, *Radix Angelicae Sinensis* (**a**) group; e and h, *Radix Astragali* + *Radix Angelicae Sinensis* (R + A) group; c, d and e were treated with 100 μg/mL of freeze-dried powders and f, g and h were treated with 250 μg/mL of freeze-dried powders. Data are presented as the mean ± SD, *n* = 3. *P < 0.05 and **P < 0.01

### Interference in IFN-γ -induced the activation of eIF2α signaling pathway in BM cells

**Fig. 9:**
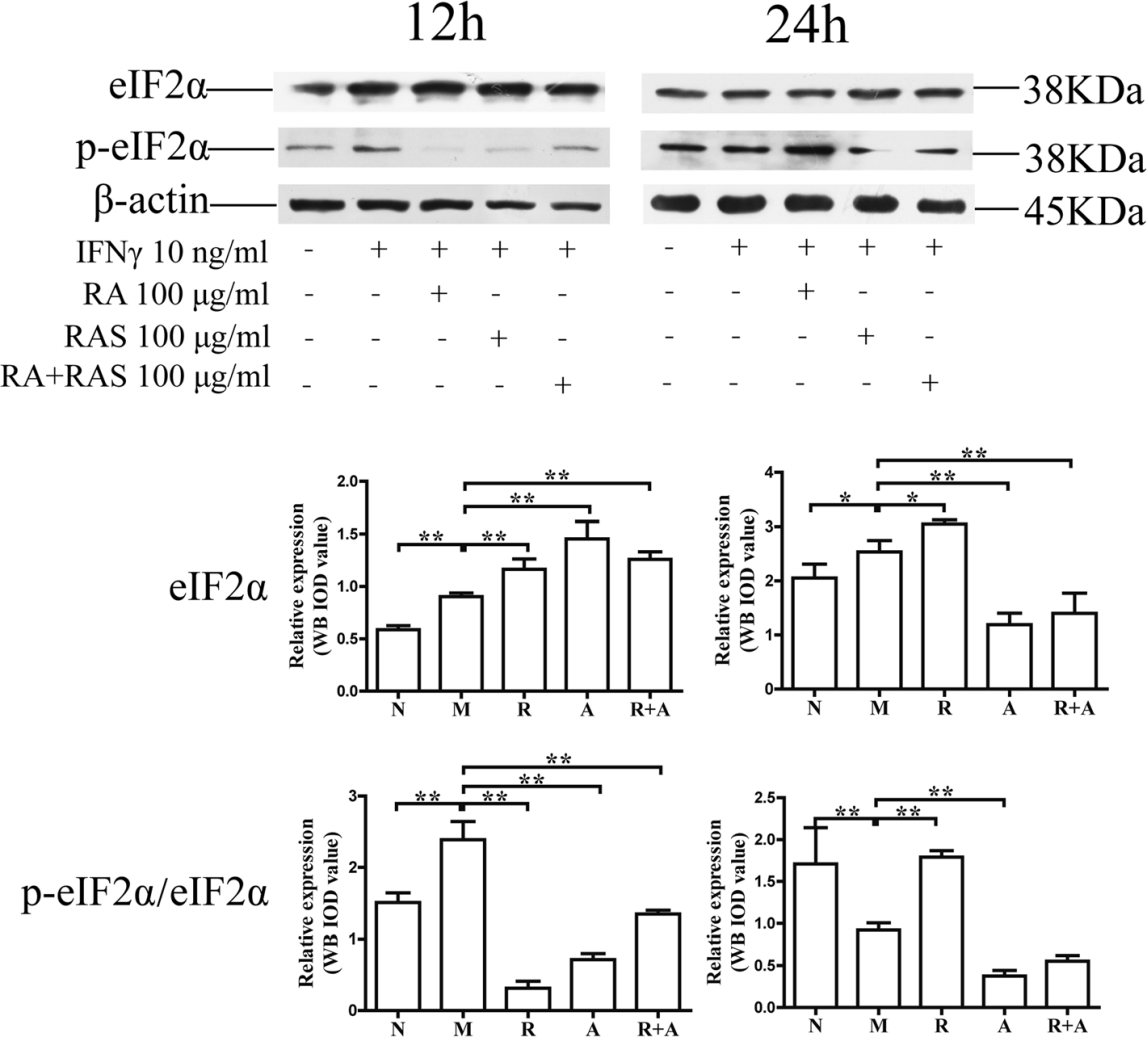
Interference in the levels of phospho-eIF2α in BM cells at 100 μg/mL of *Radix Astragali* and *Radix Angelicae Sinensis* treatment. The expression levels of eIF2α and phospho-eIF2α in BM cells were determined by Western blot analysis. β-actin was used as an internal control. The quantified results are presented in a bar chart. Data are presented as the mean ± SD, *n* = 3. *P < 0.05 and **P < 0.01

**Fig. 10:**
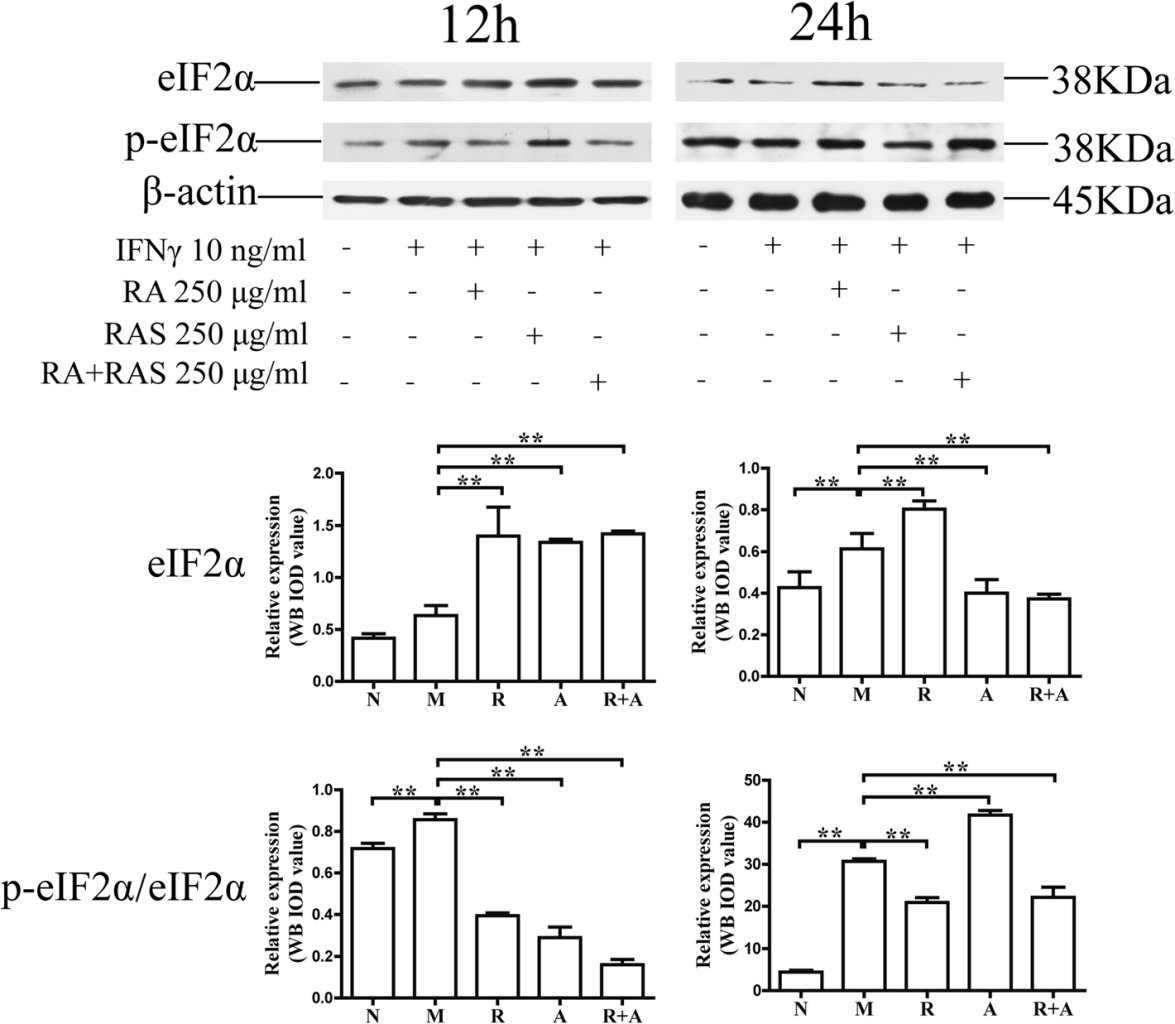
Interference in the levels of phospho-eIF2α in BM cells at 250 μg/mL of *Radix Astragali* and *Radix Angelicae Sinensis* treatment. The expression levels of eIF2α and phospho-eIF2α in BM cells were determined by Western blot analysis. β-actin was used as an internal control. The quantified results are presented in a bar chart. Data are presented as the mean ± SD, *n* = 3. *P < 0.05 and **P < 0.01

The activation of eIF2 can downregulate the protein synthesis of BM cells under condition of several stresses [29]. The levels of eIF2α and phospho-eIF2α increased significantly compared with those of the normal group (*P* < 0.01 or *P* < 0.05, respectively). The phosphorylation level of eIF2α in the treatment groups decreased significantly compared with that of the model group (*P* < 0.01). These findings indicate that both *Radix Astragali* and *Radix Angelicae Sinensis* could inhibit activation of the eIF2α signaling pathway. The synergistic effect of the two-herb combination was superior to that of a single herb alone (Figs. 9 and 10).

## Discussion

Herbal combinations, based on the herbal compatibility theory in traditional Chinese medicine (TCM), can enhance the therapeutic efficacy of a single herb [30]. Synergistic effects between *Radix Astragali* and *Radix Angelicae Sinensis* in DBT have been well illustrated [5]. Quality control of herbal combination is a precondition for cell experimental stability and reliability. Quality control of herbal combination is a precondition for cell experimental stability and reliability. HPLC-MS is an essential technology for the analysis of herbal constituents.

HPLC-ESI/MS^n^ analysis identified seven *Radix Astragali* and six *Radix Angelicae Sinensis*constituents in water extract of the herbs. Calycosin and formononetin, the major compounds in *Radix Astragali*, can promote cell adhesion [31], induce the expression of erythropoietin in kidney and liver cells [32] and regulate the activation of anti-oxidative enzymes [33]. Ferulic acid, the main component in *Radix Angelicae Sinensis*, can promote the proliferation of HSCs and hematopoietic function of bone marrow [34–36]. Based on pharmacological research, we hypothesized that the combination of *Radix Astragali* and *Radix Angelicae Sinensis* could attenuate immune attack on BM cells induced by pro-inflammatory cytokinesand the hematopoietic function of bone marrow. In this study, we also aimed to validate the synergistic effects of herbal compatibility.

Decreased numbers of IFN-γ-producing lymphocytes have been associated with hematologic improvement following immunosuppression in patients with hypoplastic myelodysplasia [1]. IFN-γ, secreted by activated T cells in the BM, also has a profound impact on the hematopoietic system. IFN-γ can suppress the formation of several hematopoietic lineages including B cells [37], erythrocytes [38], eosinophils [39] and neutrophilic granulocytes [40], resulting in increased cell death of hematopoietic progenitors by inducing cell apoptosis [41, 42]. The levels of IFN-γ and TNF-α were increased significantly in bone marrow and peripheral blood of patients with aplastic anemia. Increased IFN-γ and TNF-α can induce apoptosis of HSCs directly, promote the expression of Fas antigen on the surface of CD34^+^ cells in the bone marrow microenvironment, induce HSC apoptosis, and then inhibit the hematopoietic function of bone marrow [43, 44]. Our results showed that *Radix Angelicae Sinensis* treatment at high concentrations could increase the proliferation of HSCs in bone marrow after immune destruction. The synergistic effect of *Radix Astragali* and *Radix Angelicae Sinensis* in combination on hematopoiesis was superior to that of a single herb alone.

T lymphocytes from AA patients cocultured with bone marrow cells were able to suppress hematopoiesis in vitro [45]. Oligoclonally expanded cytotoxic T cells (targeting hematopoietic stem and progenitor cells) and activated CD8^+^ T cells were identified as the lymphocyte s subsets that inhibited hematopoiesis in the bone marrow microenvironment. Removing the lymphocytes from bone marrow could improve colony numbers in tissue culture. Specific CD8^+^CD28^−^ cell clones are expanded in AA peripheral blood, and oligo-clones recognize and induce apoptosis of autologous myeloid cells [46]. Our results indicated that both *Radix Astragali* and *Radix Angelicae Sinensis* could regulate the proliferation and differentiation of T cells. The two herbs could downregulate the levels of CD3^+^CD4^+^ T cells at low concentrations. However, the percentages of CD3^+^CD4^+^ T cells were upregulated after 24 h of treatment at high concentrations. The herbal treatment also inhibited the abnormal proliferation of CD3^+^CD8^+^ T cells. The combination of the two herbs showed greater synergistic effects compared with that of either herb alone. These effects had a significant dose-effect relationship.

T-bet binding to the IFN-γ promoter region is critical for Th1 polarization. T-bet can be upregulated in the T cells of patients with AA. T-bet binds to the promoter region of the IFN-γ gene and stimulates the protein expression of IFN-γ. CD150 combined with SLAM can bind to the signal molecule SAP and modulate the function of SLAM in IFN-γ production in hematopoietic cells [47, 48]. The freeze-dried water extract of *Radix Angelicae Sinensis* could significantly decreased the expression of T-bet after 12 h of treatment. *Radix Astragali* significantly increased the number of SLAM/SAP double-stained cells after 12 h of treatment at low concentrations. The synergistic effect of the combination of the *Radix Astragali* and *Radix Angelicae Sinensis* was better than that of other groups at high concentrations Based on these results, we considered that the combination of these two herbs could interfere with the expression of T-bet and the binding of SAP and Fyn to inhibit the function of IFN-γ, contributing to restoring the hematopoietic function of BM cells.

IFN-γ can activate IRF-1, induce the activation of PRK/eIF2 signaling pathways, interfere in gene transcription, protein synthesis and periodic cell division, and inhibit the proliferation of HSCs. Treatment with *Radix Astragali* and *Radix Angelicae Sinensis* could regulate the protein expression and phosphorylation of eIF2α. The regulatory effect of *Radix Angelicae Sinensis* at high concentrations was better than that of *Radix Astragali*. The synergistic effects of the combination of the two herbs were better than those of a single herb alone after 12 h and 24 h of treatment. These results indicated that PRK/eIF2 signaling pathways could also be one of the important targets for the synergistic hemopoietic stimulating function of the combination of *Radix Astragali* and *Radix Angelicae Sinensis*.

Compatibility of Chinese herbs, which compose TCM formulas, can achieve synergistic therapeutic effects. However, the therapeutic mechanisms and targets of compatibility of Chinese herbs have not yet been identified. Our results indicate that the therapeutic function of *Radix Astragali* focused on suppressing immune inflammation while that of *Radix Angelicae Sinensis* focused on repairing the immune destruction of BM cells. The combination of the two herbs could attenuate the immune destruction of hematopoiesis in BM cells induced by pro-inflammatory cytokines, achieving a synergetic effect based on TCM compatibility theory. The results of herbal composition analysis also provided other relevant evidence.

## Conclusions

In summary, our results indicated that the combination of *Radix Astragali* and *Radix Angelicae Sinensis* could reduce the proliferation and differentiation of effector T cells, restore the balance of the T cell immune response network and recover the hematopoietic function of HSCs. The main mechanisms likely involve inhibiting the IFN-γ-induced expression of T-bet in the bone marrow microenvironment, intervening in the activation of the eIF2 signaling pathway, attenuating immune-mediated destruction of HSCs induced by pro-inflammatory cytokines. Prolonged stimulation at high concentrations of combined *Radix Astragali* and *Radix Angelicae Sinensis* on marrow failure induced by immune attack showed improved therapeutic effect. The synergistic effect of the combination of these two herbs was superior to that of a single herb alone.

## Supplementary information

**Additional file 1** Inspection reports of Radix Astragali and *Radix Angelicae Sinensis*.

**Additional file 2: Figure. S1.** The cell viability of Bone marrow cells treated by RAS (A) and RA (B). Results were presented in a bar chart. Data were presented as mean ± SD, *n* = 3.

## Data Availability

The datasets used and/or analyzed during the current study are available from the corresponding author upon a reasonable request. All data generated or analyzed during this study are included in this published article and its supplementary information files.

## Abbreviations

AA: Aplastic anemia
BM: Bone marrow
DBT: Danggui buxue tang
HPLC-ESI/MS^n^: High-performance liquid chromatography-electrospray ionization/mass spectrometer
HSCs: Hematopoietic stem cells
IFN: Interferon
IRF: Interferon regulatory factor
OD: Optical density
RA: *Radix astragali*
RAS: *Radix angelicae sinensis*;
S.D.: Standard deviation
