# Supplementary material for: The combination of Radix Astragali and Radix Angelicae Sinensis attenuates the IFN-γ-induced immune destruction of hematopoiesis in bone marrow cells
Source: BMC Complement Altern Med. 2019 Dec 9;19:356. doi: 10.1186/s12906-019-2781-4 (PMC6902408; doi:10.1186/s12906-019-2781-4)
Supplement: Supplementary file 1 — Additional file 1 Inspection reports of Radix Astragali and Radix Angelicae Sinensis. [file 12906_2019_2781_MOESM1_ESM.pdf]

北京西单医药有限责任公司
检验报告单

检验单号：C19042902

品　　名：	黄芪	规　　格：	优质
产　　地：	内蒙	数　　量：	6190kg
编号/批号：	19042102	生产日期：	2019-04-21
检品来源：	包装车间	取样日期：	2019-04-23
检验项目：	全检	报告日期：	2019-04-29
检验依据：	《中国药典》2015 版一部以及四部		

检验项目	标准规定	检验结果
性状	应与黄芪性状相符	与黄芪性状相符
显微鉴别	应与黄芪显微特征相符	与黄芪显微特征相符
薄层鉴别	供试品色谱中，在与对照品色谱相应的位置上，日光下应显相同的棕褐色斑点；紫外光灯（365nm）下应显相同的橙黄色荧光斑点	供试品色谱中，在与对照品色谱相应的位置上，日光下显相同的棕褐色斑点；紫外光灯（365nm）下显相同的橙黄色荧光斑点
	供试品色谱中，在与对照药材色谱相应的位置上，应显相同颜色的荧光主斑点	供试品色谱中，在与对照药材色谱相应的位置上，显相同颜色的荧光主斑点
杂质	不得过 3%	0.7%
水分	不得过 10.0%	7.7%
总灰分	不得过 5.0%	3.7%
重金属及有害元素	应符合药典要求	符合药典要求
有机氯农药残留量	应符合药典要求	符合药典要求
浸出物	不得少于 17.0%	19.4%
含量测定	含黄芪甲苷($C_{41}H_{68}O_{14}$)不得少于 0.040％	0.460%
	含毛蕊异黄酮葡萄糖苷($C_{22}H_{22}O_{10}$)不得少于 0.020％	0.078%
二氧化硫残留量	不得过 150mg/kg	65mg/kg

检验结论：本品按上述检验依据检验，结果符合规定。

北京西单医药有限责任公司 饮片药检专用章

检验人：田思思　　复核人：曹金月　　审核人：佟欣

Beijing Xidan Pharmaceutical Co. Ltd Inspection Report

Inspection Sheet No.: C19042902
Latin Name: *Radix Astragali*
Place of Origin: Inner Mongolia, China
Serial Number: 19042102
Inspection Item: Full-Inspection
Inspection Standard: *Chinese pharmacopoeia 2015 edition* 1 and 4

Standard: High Quality
Production Date: April 21, 2019
Sampling Date: April 23, 2019
Report Date: April 29, 2019

Inspection Item	Standard Stipulations	Inspection Results
Character	It should be consistent with *Radix Astragali* characters	It is consistent with *Radix Astragali*
Microscopical Characteristic	It should be consistent with the microscopic characteristics of *Radix Astragali*	It is consistent with the microscopic characteristics of *Radix Astragali*
Thin Layer Chromatography Identification	The same brown spots should be seen in the sunlight on the corresponding position of the chromatogram of the reference substance and the same orange fluorescent spots in UV lamps (365nm)<br>The fluorescence main spot of the same color should be displayed in the corresponding position of the chromatogram of the control herbs.	The same brown spots appear in the sunlight at the corresponding position of the reference substance chromatography and the same orange fluorescent spots in UV lamp (365nm)<br>In the chromatogram of the sample, the fluorescence main spots of the same color appeared on the corresponding chromatogram of the control drug.
Foreign Matter	≤3.0%	0.7%
Moisture	≤10.0%	7.7%
Total Ash	≤5.0%	3.7%
Heavy Metal and Deleterious Element	It should meet the *Chinese pharmacopoeia 2015 edition* requirements	It meets the *Chinese pharmacopoeia 2015 edition* requirements
Organochlorine Pesticide Residue	It should meet the *Chinese pharmacopoeia 2015 edition* requirements	It meets the *Chinese pharmacopoeia 2015 edition* requirements
Extract	≥17.0%	19.4%
Content Determination	The content of *Astragaloside Iv* ($C_{41}H_{68}O_{14}$) ≥0.040%<br>The content of *Calycosin-7-glucoside* ($C_{22}H_{22}O_{10}$) ≥0.020%	0.460%<br>0.078%
Residual Sulfur Dioxide	≤150mg/kg	65mg/kg

Inspection Conclusion: After above inspection, the quality of this products pass the inspection.

Inspectors: Sisi Tian Checker: Jinyue Cao Verifier: Xin Tong

北京西单医药有限责任公司 快检专用章

北京西单医药有限责任公司
检验报告单

检验单号：C19051402

品　　名：	当归	规　　格：	优质
产　　地：	甘肃	数　　量：	430kg
编号/批号：	19050802	生产日期：	2019-05-08
检品来源：	包装车间	取样日期：	2019-05-10
检验项目：	全检	报告日期：	2019-05-14
检验依据：	《中国药典》2015 版一部以及四部		

检验项目	标准规定	检验结果
**性状**	应与当归性状相符	与当归性状相符
**显微鉴别**	应与当归显微特征相符	与当归显微特征相符
**薄层鉴别**	供试品色谱中，在与对照药材色谱相应的位置上，应显相同颜色的荧光斑点	供试品色谱中，在与对照药材色谱相应的位置上，显相同颜色的荧光斑点
	供试品色谱中，在与对照品色谱相应的位置上，应显相同颜色的荧光斑点	供试品色谱中，在与对照品色谱相应的位置上，显相同颜色的荧光斑点
**杂质**	不得过 3%	1.3%
**水分**	不得过 15.0%	12.2%
**总灰分**	不得过 7.0%	5.4%
**酸不溶性灰分**	不得过 2.0%	0.4%
**浸出物**	不得少于 45.0%	47.4%
**二氧化硫残留量**	不得过 150mg/kg	80mg/kg

（印章：北京西单医药有限责任公司 饮片药检专用章）

检验结论：本品按上述检验依据检验，结果符合规定。

检验人：曹金月　　复核人：田思思　　审核人：佟欣

Beijing Xidan Pharmaceutical Co. Ltd Inspection Report

Inspection Sheet No.: C19051402
Latin Name: *Radix Angelicae Sinensis*
Place of Origin: Gansu, China
Serial Number: 19050802
Inspection Item: Full-Inspection
Inspection Standard: *Chinese pharmacopoeia 2015 edition* 1 and 4

Standard: High Quality
Production Date: May 08, 2019
Sampling Date: May 10, 2019
Report Date: May 14, 2019

Inspection Item	Standard Stipulations	Inspection Results
Character	It should be consistent with *Radix Angelicae Sinensis* characters	It is consistent with *Radix Angelicae Sinensis*
Microscopical Characteristic	It should be consistent with the microscopic characteristics of *Radix Angelicae Sinensis*	It is consistent with the microscopic characteristics of *Radix Angelicae Sinensis*
Thin Layer Chromatography Identification	Fluorescence spots of the same color should appear in the sample chromatogram corresponding to the chromatogram of the control herbs	In the chromatogram of the sample, the fluorescence spots of the same color appeared on the corresponding chromatogram of the control drug.
Foreign Matter	≤3.0%	1.3%
Moisture	≤15.0%	12.2%
Total Ash	≤7.0%	5.4%
Acid-Insoluble Ash	≤2.0%	0.4%
Extract	≥45.0%	47.4%
Residual Sulfur Dioxide	≤150mg/kg	80mg/kg

Inspection Conclusion: After above inspection, the quality of this products pass the inspection.

Inspectors: Jinyue Cao Checker: Sisi Tian Verifier: Xin Tong
