# Supplementary material for: The combination of Radix Astragali and Radix Angelicae Sinensis attenuates the IFN-γ-induced immune destruction of hematopoiesis in bone marrow cells
Source: BMC Complement Altern Med. 2019 Dec 9;19:356. doi: 10.1186/s12906-019-2781-4 (PMC6902408; doi:10.1186/s12906-019-2781-4)
Supplement: Supplementary file 2 — Additional file 2: Figure. S1. The cell viability of Bone marrow cells treated by RAS (A) and RA (B). Results were presented in a bar chart. Data were presented as mean ± SD, n = 3. [file 12906_2019_2781_MOESM2_ESM.pdf]

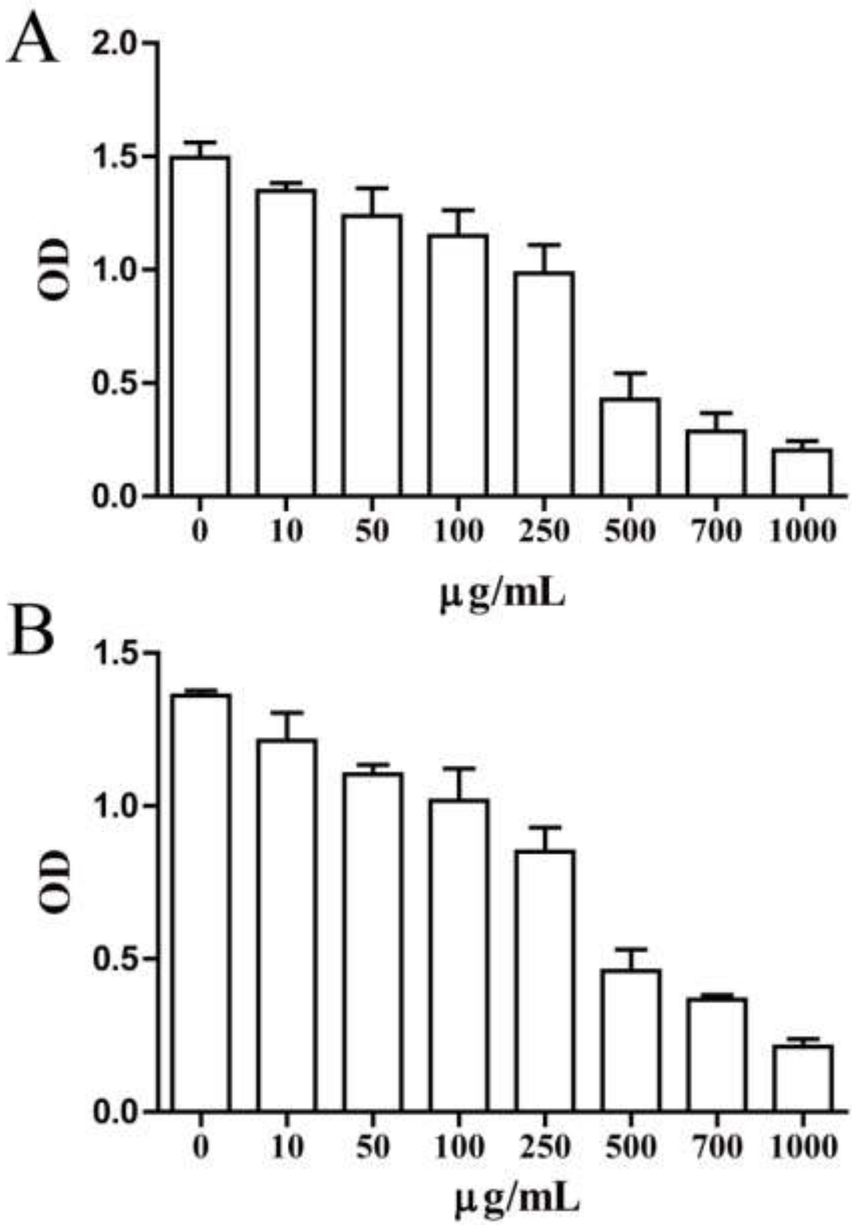

Figure S1: The cell viability of Bone marrow cells treated by RAS (A) and RA (B). Results were presented in a bar chart. Data were presented as mean ± SD, n=3.
